# *Schistosoma japonicum* Worms Alter the miRNA Expression Profile of Hepatic Stellate Cells with Potential Implications for Liver Fibrosis and Hepatocellular Carcinoma

**DOI:** 10.3390/tropicalmed11060148

**Published:** 2026-05-28

**Authors:** Haoran Zhong, Bowen Dong, Danlin Zhu, Ruiting Zhang, Yuanzhao Sun, Junhan Xiong, Liu Gao, Ke Lu, Hao Li, Zhiqiang Fu, Jinming Liu, Yamei Jin

**Affiliations:** 1 Key Laboratory of Animal Schistosomiasis of Ministry of Agriculture and Rural Affairs, Shanghai Veterinary Research Institute, Chinese Academy of Agricultural Sciences, Shanghai 200241, China; haoranzhong@shvri.ac.cn (H.Z.); dongbw131420@163.com (B.D.); 13957236028@163.com (D.Z.); zrt025@126.com (R.Z.); vr1356748202@163.com (J.X.); rw1589752587@163.com (L.G.); luke@shvri.ac.cn (K.L.); lihao@shvri.ac.cn (H.L.); fuzhiqiang@shvri.ac.cn (Z.F.); jimyliu@shvri.ac.cn (J.L.); 2 College of Animal Science and Veterinary Medicine, Shanxi Agricultural University, Jinzhong 030801, China; syz15635471166@163.com

**Keywords:** *Schistosoma japonicum*, liver fibrosis, hepatic stellate cells, microRNAs, hepatocellular carcinoma

## Abstract

Although schistosome eggs are widely recognized as the principal drivers of hepatic granulomatous inflammation and fibrosis, the independent effects of adult worms may be masked by strong egg antigen-mediated responses. This study aimed to investigate whether adult *Schistosoma japonicum* worms alter the miRNA expression profile of hepatic stellate cells and to explore the potential relevance of these changes to liver fibrosis and hepatocellular carcinoma-related processes. A non-contact Transwell co-culture system was established using paired *Schistosoma japonicum* worms or male worms and hepatic stellate cells. Male worms were additionally included to further assess worm-derived effects independent of egg production–related influences. Untreated hepatic stellate cells served as controls. Total RNA was extracted for miRNA sequencing, and differentially expressed miRNAs were identified. Target gene prediction, Kyoto Encyclopedia of Genes and Genomes pathway enrichment analysis, and validation using The Cancer Genome Atlas database were subsequently performed. Both paired worms and male worms significantly altered the miRNA expression profile of hepatic stellate cells. Several differentially expressed miRNAs were identified, among which hsa-miR-103a-3p showed relatively stable changes. Pathway enrichment analysis suggested that the potential target genes of hsa-miR-103a-3p were mainly enriched in AMP-activated protein kinase, mechanistic target of rapamycin, tumor necrosis factor, insulin signaling, and cellular senescence pathways. Further analysis using The Cancer Genome Atlas database showed that hsa-miR-103a-3p had diagnostic value in hepatocellular carcinoma and was associated with alpha-fetoprotein level, albumin level, Ishak fibrosis score, pathological stage, histological type, and tumor status. These findings suggest that adult *S. japonicum* worms may alter the miRNA expression profile of hepatic stellate cells, and that hsa-miR-103a-3p may be associated with fibrogenic responses and may have potential relevance to hepatocellular carcinoma-related processes. However, this inference is based on correlative TCGA data and does not imply a causal role in schistosomiasis-associated hepatocarcinogenesis.

## 1. Introduction

Schistosomiasis japonica is one of the most important parasitic diseases threatening human health [1,2]. Chronic infection can lead to egg deposition, persistent inflammatory responses, and hepatic tissue remodeling, ultimately resulting in liver fibrosis, portal hypertension, and even liver failure [3,4]. Hepatic injury is one of the major pathological bases of schistosomiasis, and liver fibrosis is regarded as a key determinant of disease outcome and prognosis [5,6]. Previous studies have shown that liver injury associated with *Schistosoma japonicum* infection is not simply caused by mechanical egg deposition, but rather represents a complex pathological process jointly driven by host immune responses, inflammatory mediator release, and intrahepatic cellular interactions [7,8].

Hepatic stellate cells (HSCs) are the central effector cells in the development of liver fibrosis [9]. Under physiological conditions, HSCs mainly maintain vitamin A storage and extracellular matrix homeostasis in the liver [10,11,12]. However, under stimuli such as chronic injury, inflammation, or parasitic infection, HSCs can be activated and transdifferentiate into a myofibroblast-like phenotype, secreting large amounts of extracellular matrix components and thereby promoting the progression of liver fibrosis [13]. Therefore, elucidating how schistosome-related stimuli affect the molecular regulatory network of HSCs is of great significance for understanding the mechanisms underlying schistosomiasis-associated liver fibrosis.

MicroRNAs (miRNAs) are a class of endogenous small non-coding RNAs approximately 18–25 nucleotides in length. By binding to the 3′-untranslated region (3′-UTR) of target mRNAs, they negatively regulate gene expression at the post-transcriptional level [14,15,16]. Increasing evidence has demonstrated that miRNAs participate in biological processes such as cell proliferation, apoptosis, inflammatory responses, metabolic reprogramming, and fibrosis [17,18,19,20,21]. During liver fibrosis, specific miRNAs can influence HSC activation and extracellular matrix deposition by regulating signaling pathways such as TGF-β, NF-κB, PI3K/AKT, and MAPK [22,23]. However, studies investigating the direct or indirect regulation of HSC miRNA expression by *S. japonicum* worms remain limited, and systematic comparisons of the effects induced by different worm states are still lacking [24].

Chronic liver fibrosis is widely recognized as a major predisposing factor for the development of hepatocellular carcinoma (HCC). Liver fibrosis and HCC are not isolated pathological events, but rather important stages along the continuum of chronic liver injury [25]. Persistent inflammation and a fibrotic microenvironment can significantly increase the risk of HCC, while activated HSCs not only participate in fibrogenesis but also promote tumor progression by secreting cytokines, remodeling the extracellular matrix, and shaping the tumor microenvironment [26]. Therefore, parasite-induced dysregulation of key miRNAs in HSCs may be associated not only with liver fibrosis, but also with the molecular regulation of hepatocarcinogenesis and tumor progression [27].

Based on this, in the present study, we established a Transwell co-culture system of paired *S. japonicum* worms or single male worms with HSCs. Using miRNA sequencing, we systematically analyzed changes in the miRNA expression profile of HSCs after co-culture, identified key differentially expressed miRNAs, and further performed KEGG pathway enrichment analysis and validation using the TCGA database. Through this study, we aimed to characterize the miRNA-related changes underlying the effects of *S. japonicum* worms on HSCs and to evaluate the potential biological significance of key miRNAs in liver fibrosis and HCC-related processes.

## 2. Materials and Methods

### 2.1. Ethical Considerations

All animal procedures were conducted in accordance with the regulations of the Committee for the Care and Use of Laboratory Animals at the Shanghai Veterinary Research Institute, Shanghai, China (permit no. SYXK-20160010). The experimental protocol was reviewed and approved by the Ethics and Animal Welfare Committee of the Shanghai Veterinary Research Institute, Shanghai, China (approval no. SV-20230505-03).

### 2.2. Laboratory Animals, Parasites and Infection

Specific-pathogen-free (SPF) male BALB/c mice, aged 6–8 weeks were obtained from Shanghai Jiesijie Laboratory Animal Co., Ltd. (Shanghai, China). *S. japonicum* cercariae were provided by the Shanghai Veterinary Research Institute. Infection was established by percutaneous exposure of the shaved abdominal skin to cercariae. At the specified time points, mice were anesthetized and euthanized by cervical dislocation, and paired adult worms were recovered by hepatic-portal perfusion as previously described [28]. Mated male (MM) worms were then carefully separated from the paired worms under a microscope for collection.

### 2.3. Cell Culture

LX-2, a human hepatic stellate cell line, was obtained from Boster (Wuhan, China) and cultured in DMEM (Corning, New York, NY, USA) containing 10% heat-inactivated FBS (Gibco, Shanghai, China) and 1% penicillin-streptomycin (Thermo, Shanghai, China) at 37 °C in a humidified incubator with 5% CO_2_.

### 2.4. Transwell System Establishment

BALB/c mice were infected percutaneously with approximately 100 bisexual cercariae, and adult worms were harvested at 28 days post-infection. Independent biological replicates were generated using separate batches of adult worms isolated from different infected mice. The parasites were washed three times with 20 mL PBS and maintained in prewarmed DMEM (Corning, Shanghai, China) before use. LX-2 cells (5 × 10^5^ cells/well) were seeded into the lower chamber of 12-well Transwell plates (0.4 μm PET membrane; Corning, Shanghai, China) in 1.5 mL DMEM containing 10% FBS (Corning, Shanghai, China) and 1% penicillin-streptomycin (Corning, Shanghai, China). Five pairs of adult worms or 10 mated male worms were added to the upper chamber in 900 μL fresh complete DMEM. Inserts containing unused schistosomal medium served as controls. All co-culture systems were established with at least three technical replicates and incubated at 37 °C with 5% CO_2_. During the 48-h co-culture period, worm viability was routinely monitored by light microscopy based on motility and morphological integrity. The worms remained actively motile and morphologically intact throughout the experiment, with no obvious signs of degeneration observed. After 48 h, cells were harvested for small RNA-seq.

### 2.5. Small RNA Library Preparation and Bioinformatics Analysis

Small RNA libraries were constructed using the MGIEasy Small RNA Library Prep Kit (BGI, Shenzhen, China). RNA samples were ligated with 3′ and 5′ adaptors, reverse-transcribed into cDNA, and amplified by PCR. After size selection by polyacrylamide gel electrophoresis and quality control, the libraries were denatured and circularized to generate single-stranded circular DNA, followed by digestion of residual linear DNA. DNA nanoballs were then produced by phi29 polymerase-mediated rolling circle amplification and sequenced on the G400 platform (BGI, Shenzhen, China) with an SE50 strategy. After removal of adaptor sequences and low-quality reads, clean reads were obtained, collapsed into non-redundant sequences, and mapped to reference genome (Homo sapiens: GCF_000001405.39_GRCh38.p13) using Bowtie [29]. Reads corresponding to rRNA, tRNA, snRNA, and snoRNA were identified by alignment against non-coding RNA annotation databases and subsequently removed prior to miRNA identification. The remaining reads were then used for downstream miRNA annotation and differential expression analysis. Differential expression analysis of miRNAs between groups was subsequently performed based on normalized read counts, and differentially expressed miRNAs were identified according to the thresholds of |log2 fold change| ≥ 1 and Qvalue < 0.05. Hierarchical clustering analysis was performed on the union set of differentially expressed miRNAs using the R package pheatmap (version 1.0.13) based on normalized expression data. The bioinformatic analysis software miRanda (version 3.3a) [30] was applied to predict mRNA targets of hsa-miR-103a-3p in Homo sapiens. To gain insight into the phenotypic changes, Kyoto Encyclopedia of Genes and Genomes (KEGG) [31] analysis of hsa-miR-103a-3p target genes was subsequently performed.

### 2.6. Clinical Correlation Analysis of the Cancer Genome Atlas Program (TCGA) Platform

Expression data for hsa-miR-103a-3p were obtained from the TCGA pan-cancer cohort using the Xiantao platform (accessed 20 March 2026) [32]. Correlations between hsa-miR-103a-3p expression and clinical features, including histological type, pathologic stage, tumor status and Ishak fibrosis score, were analyzed in the clinical significance module based on TCGA patient data. Welch′s one-way ANOVA (version 9.5.1) was applied for statistical comparisons. *p* values were adjusted using the Benjamini–Hochberg false discovery rate correction, and adjusted *p* < 0.05 was considered statistically significant.

### 2.7. Diagnostic Value Analysis

The diagnostic performance of hsa-miR-103a-3p in HCC was assessed by receiver operating characteristic (ROC) curve analysis using the R program (version 4.2.1) pROC package (version 1.18.0). A total of 425 samples from the TCGA cohort were included in the ROC analysis. Diagnostic accuracy was quantified by the area under the curve (AUC), where higher AUC values reflect better discriminatory ability. An AUC of 0.5–0.7 is generally regarded as low, 0.7–0.9 as moderate, and >0.9 as high diagnostic accuracy [33].

## 3. Results

### 3.1. Transwell Co-Culture with S. japonicum Worms Altered the miRNA Expression Profile of HSCs

**Figure 1:**
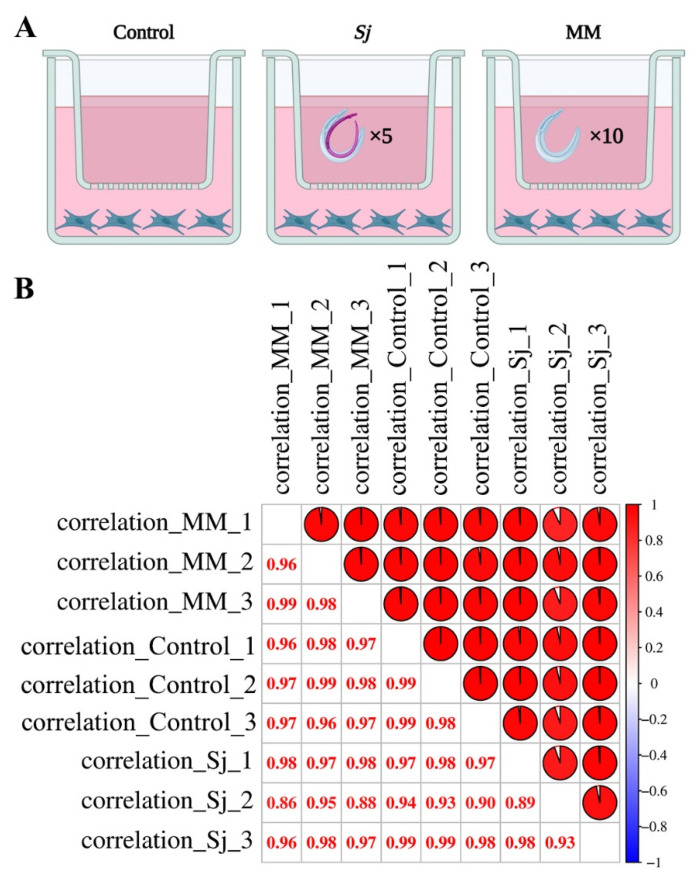
Experimental design and sample correlation analysis of small RNA sequencing. (**A**) Schematic illustration of the Transwell co-culture system. LX-2 cells were seeded in the lower chamber, while paired *S. japonicum* worms (*Sj*, 5 pairs) or mated male worms (MM, 10 worms) were placed in the upper chamber. Cells cultured with control medium served as the control group. (**B**) Correlation matrix of small RNA sequencing samples from the MM, control, and *Sj* groups.

To investigate whether *S. japonicum* adult worms could modulate the miRNA landscape of HSCs, a non-contact Transwell co-culture system was established in which paired worms (*Sj*) or male worms alone (MM) were co-incubated with HSCs, while PBS-treated cells served as the control group (Figure 1A). This experimental design allowed us to evaluate the effects of worm-derived soluble factors on HSCs while excluding direct physical contact between worms and host cells.

Correlation analysis of miRNA sequencing data demonstrated high reproducibility among biological replicates within each group. As shown in Figure 1B, the correlation coefficients among samples were consistently high, with most values approaching 1.0, indicating robust data quality and good intra-group consistency. Meanwhile, distinguishable differences were observed across treatment groups, suggesting that exposure to *S. japonicum* worms altered the overall miRNA expression pattern in HSCs.

Taken together, these findings indicate that both paired worms and male worms alone are capable of remodeling the post-transcriptional regulatory landscape of HSCs, supporting the notion that parasite-derived signals may participate in shaping the fibrogenic microenvironment through miRNA-mediated mechanisms.

### 3.2. Differential Expression Analysis Identified hsa-miR-103a-3p as a Candidate Key miRNA

**Figure 2:**
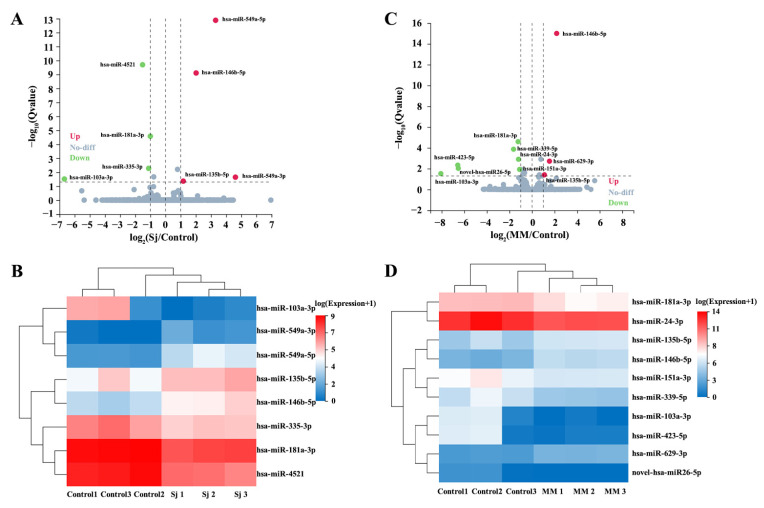
Differentially expressed miRNAs in HSCs after co-culture with *S. japonicum* worms. (**A**) Volcano plot showing differentially expressed miRNAs in the *Sj* group compared with the control group. (**B**) Heatmap of selected differentially expressed miRNAs in the *Sj* and control groups. (**C**) Volcano plot showing differentially expressed miRNAs in the MM group compared with the control group. (**D**) Heatmap of selected differentially expressed miRNAs in the MM and control groups.

To further characterize the miRNA alterations induced by parasite stimulation, differential expression analyses were performed between the *Sj* and control groups, as well as between the MM and control groups. Volcano plots revealed that multiple miRNAs were significantly dysregulated following co-culture with either paired worms or male worms alone. In the *Sj*/PBS comparison, several miRNAs were markedly upregulated or downregulated (Figure 2A and Supplementary Table S1), and a distinct set of dysregulated miRNAs was also identified in the MM/control comparison (Figure 2C and Supplementary Table S2). These results suggest that both conditions exert substantial but not entirely identical regulatory effects on HSC miRNA expression.

Hierarchical clustering analysis further demonstrated a distinguishable clustering pattern between the treatment and control groups based on differentially expressed miRNAs. As shown in Figure 2B, the *Sj* group exhibited a distinct miRNA expression pattern compared with the PBS group. Similarly, the MM and control groups could also be well discriminated by clustering analysis (Figure 2D). These results indicate that co-culture with *S. japonicum* worms not only affects individual miRNAs but also induces distinct miRNA expression patterns in HSCs.

Among the identified differentially expressed miRNAs, hsa-miR-103a-3p demonstrated one of the most pronounced and consistent expression changes across both *Sj* and MM treatments, with log_2_ fold changes of −6.68 in the *Sj*/control comparison and −8.08 in the MM/control comparison, respectively, and corresponding Q values of 0.0303 and 0.0298 (Supplementary Tables S1 and S2). Therefore, hsa-miR-103a-3p was prioritized for downstream analyses based on both the magnitude and consistency of dysregulation and its potential biological relevance to liver fibrosis-associated pathways. In addition to hsa-miR-103a-3p, several other miRNAs, including hsa-miR-549a-5p, hsa-miR-146b-5p, hsa-miR-181a-3p, hsa-miR-335-3p, hsa-miR-4521, hsa-miR-24-3p, hsa-miR-151a-3p, hsa-miR-339-5p, hsa-miR-423-5p, hsa-miR-629-3p, and novel-hsa-miR26-5p, also showed differential expression under parasite stimulation, suggesting that *S. japonicum* may regulate multiple miRNA-mediated pathways in HSCs.

Given that HSC activation is a central event in hepatic fibrosis, and persistent fibrosis/cirrhosis constitutes a major pathological basis for HCC, we reasoned that schistosome-induced miRNA alterations in HSCs may not be restricted to fibrogenesis but could also be relevant to liver tumorigenesis. On this basis, hsa-miR-103a-3p was subjected to further pathway enrichment and TCGA-based analyses.

### 3.3. KEGG Enrichment Analysis Suggested That hsa-miR-103a-3p Was Involved in Metabolism-, Inflammation-, and Cancer-Related Pathways

**Figure 3:**
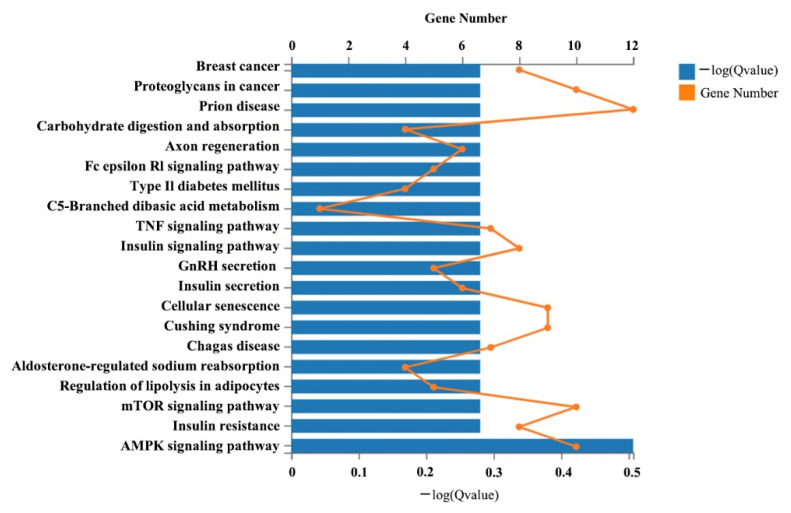
KEGG pathway enrichment analysis of candidate target genes associated with hsa-miR-103a-3p. The Gene Ratio represents the proportion of predicted hsa-miR-103a-3p target genes enriched in a given KEGG pathway relative to the total number of predicted target genes included in the KEGG enrichment analysis. The bar length indicates −log(Q value), and the orange line indicates the number of enriched genes in each pathway.

To explore the potential biological functions of hsa-miR-103a-3p, KEGG pathway enrichment analysis was performed based on its predicted target genes. As shown in Figure 3, the target genes of hsa-miR-103a-3p were mainly enriched in pathways related to AMPK signaling, insulin resistance, mTOR signaling, regulation of lipolysis in adipocytes, aldosterone-regulated sodium reabsorption, insulin secretion, GnRH secretion, insulin signaling, TNF signaling, and cellular senescence. Additional enrichment was also observed in pathways such as type II diabetes mellitus, carbohydrate digestion and absorption, proteoglycans in cancer, and breast cancer (Supplementary Table S3).

These findings indicate that hsa-miR-103a-3p may act as a multifunctional regulatory node connecting fibrogenic activation, metabolic reprogramming, and inflammation-associated signaling. Considering the well-established pathological continuum from chronic liver injury and fibrosis to HCC [34], these results provided a rationale for further evaluating the clinical significance of hsa-miR-103a-3p in liver cancer datasets.

### 3.4. TCGA Analysis Revealed That hsa-miR-103a-3p Was Closely Associated with HCC Development and Clinicopathological Characteristics

**Figure 4:**
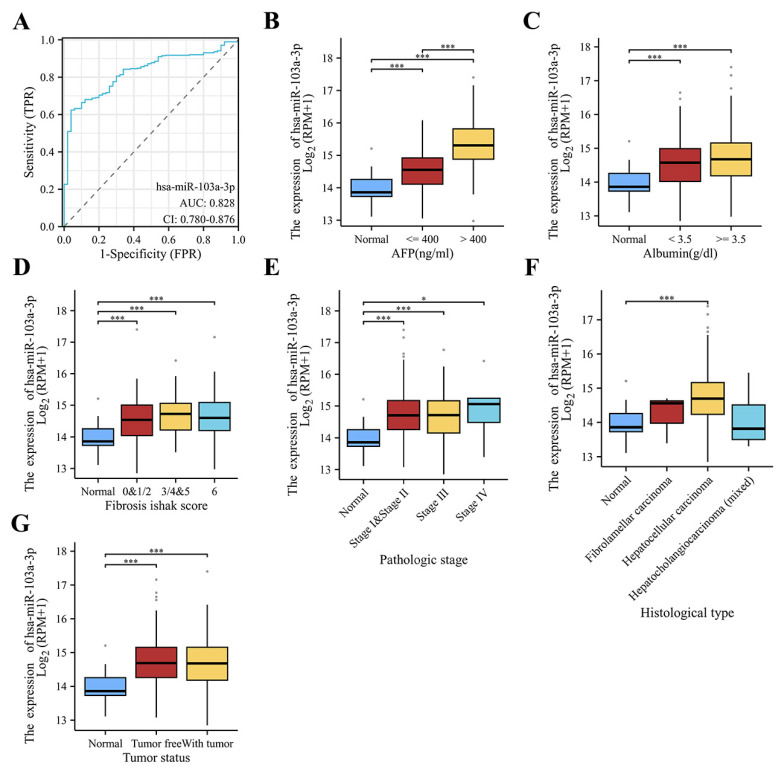
Clinical significance of hsa-miR-103a-3p in hepatocellular carcinoma based on TCGA data. (**A**) ROC curve evaluating the diagnostic performance of hsa-miR-103a-3p in HCC. (**B**) Association between hsa-miR-103a-3p expression and serum AFP level. (**C**) Association between hsa-miR-103a-3p expression and albumin level. (**D**) Association between hsa-miR-103a-3p expression and Ishak fibrosis score. (**E**) Association between hsa-miR-103a-3p expression and pathological stage. (**F**) Association between hsa-miR-103a-3p expression and histological type. (**G**) Association between hsa-miR-103a-3p expression and tumor status. Data are presented as log2 (RPM + 1). Statistical significance is indicated as * *p* < 0.05 and *** *p* < 0.001.

To further assess the potential clinical relevance of hsa-miR-103a-3p in liver malignancy, its expression pattern and clinicopathological associations in the TCGA database was analyzed. ROC curve analysis demonstrated that hsa-miR-103a-3p had a good discriminatory ability for distinguishing HCC tissues from normal liver tissues, with an AUC of 0.828 (95% CI: 0.780–0.876) (Figure 4A), indicating its potential diagnostic value.

Stratified analyses further showed that the expression of hsa-miR-103a-3p was significantly associated with multiple clinicopathological variables. Compared with normal tissues, hsa-miR-103a-3p expression was elevated in HCC samples and was further increased in patients with higher AFP levels (Figure 4B). Significant differences were also observed among subgroups stratified by albumin level (Figure 4C), suggesting a potential relationship between hsa-miR-103a-3p and liver functional status.

Importantly, hsa-miR-103a-3p expression was significantly associated with Ishak fibrosis score (Figure 4D), indicating that this miRNA may be linked not only to tumor status but also to the fibrotic background of the liver. Moreover, significant differences in hsa-miR-103a-3p expression were observed across pathological stages (Figure 4E), histological subtypes (Figure 4F), and tumor status categories (Figure 4G), further suggesting its involvement in HCC progression and biological heterogeneity.

Taken together, these data indicate that hsa-miR-103a-3p is not only a schistosome-responsive miRNA identified in HSCs, but also a clinically relevant molecule in HCC. Taken together, these data indicate that hsa-miR-103a-3p is a schistosome-responsive miRNA identified in HSCs and is also associated with clinicopathological features in HCC based on TCGA data. However, this association should be interpreted cautiously, as the TCGA cohort comprises HCC cases of heterogeneous etiologies rather than schistosomiasis-specific cases, and therefore does not provide direct evidence for a causal role in schistosomiasis-associated hepatocarcinogenesis.

## 4. Discussion

This study analyzed the effects of paired *S. japonicum* worms and single male worms on the miRNA expression profile of HSCs using a non-contact Transwell co-culture system. The obtained results suggested that both paired worms and single male worms may alter the miRNA expression pattern of HSCs, indicating that soluble molecules, EVs, or paracrine-like signals released by the worms may induce distinct molecular responses in host cells in the absence of direct contact. From the perspective of changes in host endogenous miRNAs, this phenomenon suggests that, in *S. japonicum*-associated liver disease, in addition to the classical egg-driven granuloma-fibrosis axis, adult worms may also potentially participate in the regulation of the hepatic microenvironment [24,35].

It should be noted that selecting worms rather than eggs as the stimulatory factor does not negate the central pathogenic role of eggs in schistosomal liver fibrosis. A large body of evidence has demonstrated that eggs and their antigens are key factors driving granuloma formation, Th2-biased immune responses, HSC activation, and collagen deposition, and the “egg-driven pathogenesis” framework is already well established [13]. However, under conditions lacking egg deposition and a strong egg-antigen background, whether worm-derived soluble signals are sufficient to independently induce miRNA reprogramming in HSCs remains a question of interest [24]. However, under conditions lacking egg deposition and a strong egg-antigen background, whether worm-derived soluble signals are sufficient to independently induce miRNA reprogramming in HSCs remains a question of interest. This issue has independent research significance, because adult worms reside for prolonged periods in the host portal and mesenteric venous systems, where they continuously interact with the host blood environment and release proteins, lipids, and small RNAs through excretory-secretory products (ESPs) or EVs [24]. In addition, studies have shown that prolonged single-sex worm infection can also lead to liver fibrosis, further suggesting that parasite-host molecular communication may persist even in the absence of egg deposition or during the pre-oviposition stage [7,36,37,38].

Existing studies suggest that adult worms may not merely serve as a prerequisite for egg production, but may also directly participate in the regulation of the host pathological microenvironment through their secretory products [39]. One study reported that EVs derived from adult *S. japonicum* worms can be internalized by HSCs and deliver parasite miRNAs into host cells, thereby affecting the Col1α2/TGF-β/Smad-related pathway, suggesting that worm-derived molecules may be involved in the regulation of liver fibrosis [35]. Another study observed increased expression of α-SMA, Col1α1, and Col3α1 in the periportal region in a single male worm infection model, and further found in vitro that worms could alter the transcriptome and small RNA expression profile of HSCs, indicating that, even in the absence of eggs, the worms themselves retain the potential to induce fibrosis-related molecular remodeling [39]. Relevant reviews have also proposed that single-sex schistosome infection, due to the absence of egg deposition, may facilitate the identification of the independent effects of adult worms on host tissues [24,38,40].

Another factor to consider is that the effects of eggs or soluble egg antigens (SEA) on HSCs are not unidirectionally pro-fibrotic, but rather somewhat complex [41,42,43]. Previous studies have shown that *S. japonicum* SEA can induce apoptosis of LX-2 cells and inhibit their activation by downregulating Akt and upregulating p53-dependent DR5, and can also promote senescence of activated HSCs through the STAT3/p53/p21 pathway while reducing the expression of α-SMA and procollagen I [42]. These findings suggest that if eggs or SEA are directly used to treat HSCs, the resulting miRNA changes may more likely reflect egg-antigen-induced stress, apoptosis, or senescence programs, thereby making it difficult to distinguish the independent regulatory effects of worm-derived signals on HSCs. Therefore, the worm-based Transwell co-culture design is more suitable for investigating the effects of worm-derived soluble factors on the miRNA expression profile of HSCs, and should be regarded as a complement to, rather than a substitute for, the classical egg-based model. Compared with previous egg-focused studies, which mainly emphasized granuloma formation, immune polarization, and fibrosis driven by egg antigens or SEA, the present study focused on worm-derived soluble signals under egg-free conditions. Therefore, the observed miRNA changes may better reflect the independent contribution of adult worms to HSC molecular remodeling rather than secondary responses induced by egg-associated inflammation or tissue injury.

Among all differentially expressed miRNAs, hsa-miR-103a-3p emerged as a candidate of particular interest. It exhibited a relatively stable differential expression pattern in HSCs after worm stimulation. KEGG enrichment analysis showed that its potential target genes are mainly involved in pathways such as AMPK, mTOR, TNF, insulin signaling, and cellular senescence. These pathways are closely associated with HSC activation, chronic inflammation, metabolic imbalance, and tumorigenesis, suggesting that hsa-miR-103a-3p may occupy a relatively important position in the process linking schistosome stimulation, HSC molecular responses, and fibrotic microenvironment remodeling. Since miRNAs generally function through post-transcriptional repression of target mRNAs [14], the observed downregulation of hsa-miR-103a-3p after worm stimulation may relieve inhibitory effects on fibrosis-related target genes and thereby contribute to the activation of pathways associated with HSC activation, chronic inflammation, metabolic dysregulation, and fibrotic remodeling. Although the direct downstream targets of hsa-miR-103a-3p in HSCs remain insufficiently characterized, the enrichment of its predicted target genes in AMPK, mTOR, TNF, and senescence-related pathways suggests that decreased hsa-miR-103a-3p expression may participate in the molecular processes associated with hepatic fibrosis progression. To further explore the potential clinical relevance of hsa-miR-103a-3p beyond the in vitro HSC model, we extended the analysis to TCGA HCC cohorts. The results suggested that hsa-miR-103a-3p showed moderate diagnostic discriminatory value in HCC and is associated with AFP, albumin, Ishak fibrosis score, pathological stage, histological type, and tumor status. However, it should be emphasized that these findings are based on correlation analyses in TCGA cohorts and therefore indicate associations rather than causal relationships between hsa-miR-103a-3p expression and HCC-related clinicopathological features. Importantly, the TCGA cohort used in this study does not specifically include schistosomiasis-associated HCC cases, and thus these results should be regarded as providing general contextual support for the relevance of hsa-miR-103a-3p in HCC, rather than direct evidence linking this miRNA to parasite-driven liver cancer. In total, these findings suggest that the significance of this miRNA may not be limited to the fibrosis stage, but may also extend to the continuous pathological progression from chronic injury to fibrosis and tumorigenesis. Given that HSCs are both the core effector cells in liver fibrosis and important components of the tumor microenvironment, extending the analysis from HSCs to HCC cohorts appears to be logically reasonable. Supporting this, recent studies in other cancer types have highlighted the prognostic and molecular relevance of hsa-miR-103a-3p. For instance, comprehensive transcriptome and miRNome profiling in metachronous colorectal liver metastasis identified hsa-miR-103a-3p as associated with distinct molecular subtypes and potential prognostic outcomes, suggesting its involvement in metastatic progression and microenvironmental remodeling [44]. Similarly, bioinformatics analyses in head and neck squamous cell carcinoma have indicated that hsa-miR-103a-3p may serve as a prognostic biomarker, further supporting its broader functional relevance in linking cellular regulatory networks to disease progression [45]. Given its association with fibrosis-related signaling pathways and HCC clinicopathological characteristics, hsa-miR-103a-3p may serve not only as a candidate biomarker for disease progression but also as a potential mechanistic probe for understanding parasite-associated hepatic remodeling. Future gain- and loss-of-function studies may help determine whether targeting hsa-miR-103a-3p could provide therapeutic value in fibrosis-related liver disease.

In addition to hsa-miR-103a-3p, several other differentially expressed miRNAs with potential biological significance were also identified. Among them, hsa-miR-146b-5p has been reported to be upregulated in fibrotic liver tissues and activated HSCs, and to promote HSC activation by targeting HIPK1 [46]; in HCC, however, it has also been reported to exert tumor-suppressive effects through the TRAF6/Akt axis, suggesting that its role may vary across different pathological settings [47]. Studies related to hsa-miR-181a have shown that it is involved in TGF-β-induced fibrosis and can regulate HSC activation and inflammatory responses through the MALAT1/miR-181a/TLR4/NF-κB axis [48,49]. Therefore, the changes in hsa-miR-181a-3p observed in this study may be closely associated with HSC activation. In addition, studies on hsa-miR-24-3p, hsa-miR-151a-3p, and hsa-miR-423-5p have focused more on HCC, vesicle-mediated communication, and therapeutic resistance, linking them to tumor cell proliferation and migration, formation of the pre-metastatic liver microenvironment, and sorafenib resistance, respectively [50,51,52]. In contrast, direct evidence for the involvement of hsa-miR-335-3p, hsa-miR-549a-5p, hsa-miR-4521, and hsa-miR-629-3p in liver fibrosis and other parasitic diseases remains limited, and these miRNAs may therefore be better regarded as novel candidates worthy of further investigation. Overall, the coordinated changes in these differentially expressed miRNAs suggest that the regulation of HSCs by *S. japonicum* worms may depend on a multi-miRNA regulatory network rather than on a single key miRNA alone.

From the perspective of the existing literature, previous reports have reported the effects of schistosome-derived miRNAs from worms entering HSCs via EVs and modulating fibrosis-related pathways [35]. Other studies have suggested that worm stimulation can alter the host transcriptome and small RNA profile of HSCs, and have shown increased fibrosis-related indicators in liver tissue under single male worm infection conditions [39]. On this basis, the responsive remodeling of host endogenous miRNAs after worm stimulation also warrants attention. These findings suggest that the effects of adult worms on HSCs may not only involve the delivery of schistosome-derived molecules, but may also include the induction of reprogramming in the host′s own miRNA regulatory network through worm secretory products. This phenomenon may contribute to a deeper understanding of the multi-layered molecular basis of parasite-host interactions.

Several limitations of this study should also be acknowledged. First, although the Transwell co-culture system is useful for highlighting the effects of worm-derived soluble signals, it cannot fully recapitulate the complex pathological setting in vivo, which involves eggs, immune cells, hepatocytes, cholangiocytes, and the blood flow environment [53]. In addition, species differences should be considered, as adult *S. japonicum* worms were obtained from infected mice, whereas LX-2 is a human hepatic stellate cell line. This interspecies experimental setting may not fully reflect host-parasite interactions occurring in human schistosomiasis. Second, this study emphasizes the possibility that adult worms independently regulate HSCs; therefore, the findings are more appropriately viewed as a complement to the classical “egg-driven pathogenesis” framework rather than a replacement for it. Third, the functional interpretation of hsa-miR-103a-3p and other candidate miRNAs currently relies mainly on bioinformatics and database analyses, and lacks sufficient gain- and loss-of-function experimental validation. Another limitation is that the present analysis focused on comparisons of *Sj* versus control and MM versus control, whereas a direct statistical comparison between the *Sj* and MM groups was not performed. Therefore, although both paired worms and mated male worms induced marked alterations in the miRNA expression profile of HSCs, the current data cannot determine which miRNAs are specifically associated with sex-dependent or pairing-dependent effects. The biological differences between paired worms and male worms may be relevant, because pairing status, worm sex, and reproductive activity could influence the composition of worm-derived soluble products or extracellular vesicles. Future studies incorporating direct *Sj* versus MM comparisons, as well as separate analyses of male worms, female worms, and paired worms, will be necessary to clarify whether specific miRNA changes in HSCs are driven by sex-, pairing-, or egg-production-associated factors. Finally, the clinical relevance evidence provided by TCGA is derived from HCC cohorts rather than schistosomiasis-specific cohorts, and is therefore better suited to supporting correlations across disease stages rather than directly demonstrating causality in the context of *S. japonicum* infection.

In summary, our findings suggest that *S. japonicum* worms, particularly the soluble signals released under non-contact conditions, may induce significant alterations in the miRNA expression profile of HSCs. This phenomenon suggests that, in *S. japonicum*-associated liver disease, in addition to the classical egg-driven granuloma-fibrosis process, adult worms themselves may also participate in the regulation of the host hepatic microenvironment. hsa-miR-103a-3p represents one of the more representative key differentially expressed miRNAs, with potential functions involving metabolism, inflammation, and tumor-related pathways, and it is associated with multiple clinicopathological features of HCC. Meanwhile, other differentially expressed miRNAs, including hsa-miR-146b-5p, hsa-miR-181a-3p, hsa-miR-549a-5p, hsa-miR-24-3p, and hsa-miR-151a-3p, also suggest that adult worms may influence HSC biological behavior through coordinated actions of multiple miRNAs. These findings provide new clues for understanding the molecular basis of *S. japonicum*-associated liver fibrosis and suggest a potential link to tumor-related processes. However, further studies, particularly in schistosomiasis-specific clinical cohorts and functional experimental systems, are required to validate the causal role of these miRNAs in hepatocarcinogenesis.

## Data Availability

Sequencing data for small RNA-seq and RNA-seq have been deposited to Sequence Read Archive at the National Center for Biotechnical Information under accession number PRJNA1176348. Further inquiries can be directed to the corresponding author.

## Supplementary Materials

The following supporting information can be downloaded at: https://www.mdpi.com/article/10.3390/tropicalmed11060148/s1, Table S1: Differentially expressed miRNA from *Sj*/PBS; Table S2: Differentially expressed miRNA from MM/PBS; Table S3: KEGG enrichment analysis for hsa-miR-103a-3p target genes.
